# Urban environmental monitoring and health risk assessment introducing a fuzzy intelligent computing model

**DOI:** 10.3389/fpubh.2024.1357715

**Published:** 2024-06-05

**Authors:** Weijia Wang, Xin Guan, Xiaoyan Peng, Zeyu Wang, Xinyi Liang, Junfan Zhu

**Affiliations:** ^1^School of Information Technology, Deakin University, Geelong, VIC, Australia; ^2^Guangzhou Xinhua University, Dongguan, China; ^3^School of Government, Sun Yat-sen University, Guangzhou, China; ^4^School of Public Administration, Guangzhou University, Guangzhou, China; ^5^Guangdong Finance and Trade Vocational College, Qingyuan, China

**Keywords:** fuzzy intelligent computing model, multi-media environmental monitoring, credibility analysis, correlation analysis, health risk

## Abstract

**Introduction:**

To enhance the precision of evaluating the impact of urban environments on resident health, this study introduces a novel fuzzy intelligent computing model designed to address health risk concerns using multi-media environmental monitoring data.

**Methods:**

Three cities were selected for the study: Beijing (*B* City), Kunming (*K* City), and Wuxi (*W* City), representing high, low, and moderate pollution levels, respectively. The study employs a Fuzzy Inference System (FIS) as the chosen fuzzy intelligent computing model, synthesizing multi-media environmental monitoring data for the purpose of urban health risk assessment.

**Results:**

(1) The model reliably estimates health risks across diverse cities and environmental conditions. (2) There is a positive correlation between PM2.5 concentrations and health risks, though the impact of noise levels varies by city. In cities *B*, *K*, and *W*, the respective correlation coefficients are 0.65, 0.55, and 0.7. (3) The Root Mean Square Error (RMSE) values for cities *B*, *K*, and *W*, are 0.0132, 0.0125, and 0.0118, respectively, indicating that the model has high accuracy. The R^2^ values for the three cities are 0.8963, 0.9127, and 0.9254, respectively, demonstrating the model’s high explanatory power. The residual values for the three cities are 0.0087, 0.0075, and 0.0069, respectively, indicating small residuals and demonstrating robustness and adaptability. (4) The model’s p-values for the Indoor Air Quality Index (IAQI), Thermal Comfort Index (TCI), and Noise Pollution Index (NPI) all satisfy *p* < 0.05 for the three cities, affirming the model’s credibility in estimating health risks under varied urban environments.

**Discussion:**

These results showcase the model’s ability to adapt to diverse geographical conditions and aid in the accurate assessment of existing risks in urban settings. This study significantly advances environmental health risk assessment by integrating multidimensional data, enhancing the formulation of comprehensive environmental protection and health management strategies, and providing scientific support for sustainable urban planning.

## 1 Introduction

With the acceleration of global urbanization, rapid urban expansion has become one of the significant challenges faced by contemporary society (1, 2). Economic resilience levels in coal-dependent and traditional cities have shown fluctuations and upward trends. However, this trend is accompanied by the exacerbation of environmental issues such as air pollution, noise, and the further deterioration of the Urban Heat Island (UHI) effect. The continuous emission of harmful gasses such as fine Particulate Matter (PM) in cities has intensified air pollution, posing a severe threat to the respiratory and cardiovascular systems of humans. Simultaneously, the UHI effect leads to increased temperatures within cities, hindering natural air circulation and imposing dual pressures of air pollution and heat stress on residents (3). High population density and traffic activities exacerbate noise pollution, directly impacting residents’ quality of life and mental and physical health. During walking activities in urban environments, adverse sensory and environmental factors become key obstacles affecting individuals’ satisfaction with comfort, thereby limiting residents’ daily activities. Sensory impacts under hot conditions are even more pronounced, leading to psychological irritability and unrest. Consequently, residents may adopt avoidance behaviors, steering clear of spaces lacking vegetation to obtain optimal shading effects. Additionally, adaptive physiological regulation strategies in dynamic environments passively concentrate populations into low-density enclosures, resulting in excessive waste of unit area from the health perspective. The current study demonstrates that urban pilot policies based on a low-carbon perspective can generate positive and complex effects on urban economic development and environmental quality (4–6). Therefore, urban development planning and design phases must thoroughly consider outdoor thermal comfort, air quality, and noise pollution. Against this backdrop, research on urban multi-medium environmental monitoring and health risk assessment becomes particularly urgent to effectively address environmental and health challenges in urban development.

Urban development is closely intertwined with environmental factors. To achieve a comprehensive understanding of various environmental factors for urban development planning, it is essential to conduct multi-medium monitoring in advance. In recent years, an increasing number of scholars have employed Fuzzy Inference Systems (FIS) as a technical tool to extract useful information from multiple urban data sources by handling fuzzy and uncertain information. By analyzing the complex relationship between urban environmental factors and the health status of residents, predictions can be made about the impact of environmental pollution on human living conditions while achieving monitoring objectives. Zeinalnezhad et al. (7) utilized an Adaptive Neuro-FIS (AN-FIS) to model time series data of key pollutants, aiming to enhance the accuracy of daily pollutant forecasts. The study revealed that traditional time series forecasting models have limitations in dealing with nonlinear and complex components, whereas the AN-FIS can more accurately predict pollutant trends. For pollutants such as CO, SO_2_, O_3_, and NO_2_, the determination coefficients of the AN-FIS were 0.8686, 0.8011, 0.8350, and 0.7640 respectively, compared to 0.8445, 0.8001, 0.7830, and 0.7602, respectively, for the semi-experimental model. Furthermore, the study indicated that the AN-FIS performed more accurately in predicting time series data (7). Shelton et al. (8) investigated seasonal variations in air pollutants such as PM2.5 and PM10, NO, CO, O_3_, and SO_2_ in two cities, Colombo and Kandy, Sri Lanka, and their relationships with meteorological variables. It showed that except for O_3_, other pollutants exhibited two peaks during the day, aligning with urban traffic congestion. Additionally, pollutant concentrations showed significant differences between different seasons (*p* = 0.013 < 0.05). Wind speed and direction played significant roles in influencing pollutant concentrations and were independent of seasons. These results contributed to the formulation of air pollution control strategies tailored to specific seasons to reduce occurrences of air pollution (8). Bressane et al. (9) employed artificial intelligence methods as auxiliary tools, focusing on the FIS due to its ability to handle inherent uncertainties in complex processes, aiming to provide optimal performance. Through analysis using the forest inventory database of Southern Santa Catarina State, Brazil, various machine learning methods were trained using 10-fold cross-validation. Results demonstrated that FIS exhibited the highest performance, with an accuracy of 98.3%, a kappa value of 0.93, and no significant difference from expert classification (*p* = 0.976). The significance level of the study was 0.05. Therefore, it concluded that FIS has potential application prospects in classifying successional stages in subtropical Atlantic forests, which can substantially influence the guidance and decision-making process for forest logging authorization and corresponding compensation measures (9). Saini et al. (10) explored the significant impact of indoor air quality on residents’ comfort, productivity levels, health, and well-being, proposing a new application of the FIS in PM10 concentration prediction based on Particle Swarm Optimization (PSO) and Genetic Algorithm Optimization. Experimental data demonstrated that the optimized FIS exhibited better performance in predicting indoor air quality, with prediction error indicators including Mean Squared Error (MSE) = 4.3656, Mean Absolute Error (MAE) = 1.9351, Mean Absolute Percentage Error (MAPE) = 9.633%, and Root Mean Squared Error (RMSE) = 2.0894. Further improvement in RMSE was achieved with the optimized FIS-PSO = 1.0746 and FIS-GA (Genetic Algorithm) = 0.998. Consequently, the optimized FIS could serve as the foundation for real-time air quality prediction systems, contributing to enhancing the health and well-being of building occupants (10). Samani et al. (11) designed a Collaborative Space Decision Support System (SDSS) based on the Fuzzy Best-Worst Method (F-BWM) algorithm to aid in understanding and detecting the fairness of health services across different locations. SDSS evaluated the equality of health service access in different geographic regions through geographical information system data monitoring and multi-criteria decision analysis methods. The results indicated that in areas with the best performance in terms of fairness of health services, the highest score obtained by SDSS was 0.38%, while the lowest score was 0.28%. In regions with the worst performance in terms of fairness, over 70% of the areas showed varying degrees of fairness. Additionally, the calculated result of SDSS for the two-sample *t*-test was 2.5, with a critical value of approximately 1.676 < 2.5 at a significance level of 0.05. This demonstrated significant differences in scores between different regions assessed by SDSS at the 0.05 significance level, indicating certain reliability of the system in evaluating and monitoring the spatial distribution of medical service fairness. This suggested that cities could allocate medical resources more scientifically, ensure resources were directed toward areas with the highest demand, improve the fairness and coverage of health services, and effectively meet people’s medical needs, thereby appropriately reducing urban health risks (11). Sarkheil et al. (12) designed a Fuzzy Health Improvement System based on FIS and proposed two novel Fuzzy Radon Hazard Indexes (FRHI) to assess statistical radon environmental health risks and address relevant risk issues. The output index of FRHI ranged from 0 (no risk) to 100 (highest risk level). The study measured radon concentrations in urban buildings and created natural radon and emission zoning maps using ArcGIS software to identify key areas for excellent control to reduce health risks from natural radon. The study showed that the initial fuzzy level of FRHI average was 60.1, indicating considerable risk; while the maximum FRHI level after 48 h was 44.8, showing a significant decrease in risk. This demonstrated the practicality and effectiveness of the method, providing innovative fuzzy methods for random radon risk assessment. Additionally, FIS could provide reliable data analysis and monitoring support in such environmental health risk assessments, aiding in improving urban air quality and providing better-quality living spaces for health (12).

The aforementioned studies illustrate that traditional models have limitations in dealing with complex nonlinear relationships and uncertainties, while AI-based methods, particularly FIS, offer certain advantages in addressing issues such as air quality monitoring, pollutant prediction, and service equity assessment. FIS demonstrates high accuracy and reliability in prediction, classification, and evaluation, providing valuable references for urban environmental monitoring, health risk assessment, and resource allocation. However, these methods still face challenges, including computational complexity in model training and parameter optimization processes, as well as insufficient cross-medium comprehensive analysis and quantification of integrated health impacts. To address these issues, this study utilizes FIS to analyze and predict urban environmental data through the fuzzification of input data and rule definition, employing autoregressive integration and logistic regression models. Specifically, by collecting and organizing time-series data, establishing FIS trees, and fuzzifying various environmental monitoring data, optimizing model parameters, and ultimately evaluating and predicting urban health risks. The aim of this study is to overcome the limitations of traditional single data sources, improve the accuracy and comprehensiveness of evaluation, quantify multiple data sources into evaluable indicators, establish a more complete evaluation system, and provide deeper insights into urban environmental management and health risk warning.

## 2 Introducing the fuzzy intelligent computing model for urban multi-media environmental monitoring and health risk assessment analysis method

### 2.1 Research methods

This study selects three cities, namely Beijing (*B* City), Kunming (*K* City), and Wuxi (*W* City), which represent different environmental characteristics, representing high pollution, clean, and moderate pollution levels, respectively. A model for urban health risk assessment is constructed by employing a FIS as the computational model and integrating environmental monitoring data from multiple media, including air quality, water quality, soil quality, and noise levels (13, 14). The analysis of urban multi-medium environmental monitoring and health risk assessment using fuzzy intelligent computing models is divided into three parts: (1) Credibility Assessment, involving the evaluation of the credibility of fuzzy outputs to discern the confidence level associated with assessment outcomes. (2) Correlation Analysis, which entails the examination of the correlation between environmental monitoring data and health data to identify the pivotal factors exerting the most significant impact on health. (3) Applicability Analysis, focusing on the comparative assessment of results across diverse cities to validate the model’s robustness and adaptability under varied environmental conditions. The study employed historical monitoring data for the purpose of training and optimization, ensuring the precise modeling of the intricate relationship between the tangible environment and health parameters (15, 16). Through the consideration of fuzzy relationships among various environmental factors, the study achieved a comprehensive evaluation of health risks (17–20).

### 2.2 Research sample

In accordance with the National Urban Air Quality Report, released by the Chinese Ministry of Ecology and Environment in September 2022, the present study strategically opted for the inclusion of three diverse cities—namely, *B* City, *K* City, and *W* City—each characterized by distinct regional attributes and varying air quality statuses. The rationale for this selection is outlined as follows. The *National Urban Air Quality Report* indicates that there is currently no clear standard for determining the comprehensive index of environmental air quality to assess urban pollution levels. However, the Chinese Ministry of Ecology and Environment stated in the report that the comprehensive index of environmental air quality is a dimensionless index describing the overall condition of urban environmental air quality. It comprehensively considers the pollution levels of six pollutants: SO_2_, NO_2_, PM10, PM2.5, CO, and O_3_. A higher value of the comprehensive index of environmental air quality indicates a heavier overall pollution level. To select sample areas with significant differences in environmental levels during the study, statistics are conducted on the comprehensive index scores of 168 cities mentioned in this report. Specifically, after compiling the report content, it is found that the report calculates the comprehensive index for 168 cities in China, with the overall numerical range of the comprehensive index being 1.28–5.06. Among them, there is only one city, Lhasa, with a comprehensive index score below 2, and only one city, Zibo, with a comprehensive index score above 5. Therefore, when dividing pollution levels, these two cities with significant differences are not considered. Combining the rankings of all cities, the study categorizes cities with a comprehensive index of 2.30 as cities with relatively high pollution levels, as their values are close to the upper limit. Cities with a comprehensive index of 3.04 are categorized as relatively clean cities, as their values are close to the median. Cities with a comprehensive index of 3.92 are categorized as cities with moderate pollution levels, as their values fall between the higher and median values.

*B* City, serving as an economic epicenter, grapples with pronounced air pollution challenges, emblematic of a large urban center characterized by elevated pollution levels. The inclusion of *K* City, distinguished by its relatively favorable air quality, serves as a comparator to validate the model’s applicability within an environment marked by comparatively cleaner air. *W* City, positioned with a moderate air quality ranking, is incorporated into the model validation process to augment the generalizability of the research findings. Given the economic developmental and environmental dissimilarities in the eastern region, this city selection strategy systematically encompasses various pollution levels and regional scenarios, ensuring a comprehensive representation that yields extensive and comparable outcomes for the study. The Urban Air Quality Index for the three cities is illustrated in Table 1.

**Table 1:** Partial city urban air quality index.

Cities	Ranking	Total number of cities	Composite index (The larger the comprehensive index value, the heavier the comprehensive pollution level)
*K* City	3	168	2.3
*W* City	44	168	3.04
*B* City	115	168	3.92

To ensure the scientific credibility of this study, standardized sampling methods are employed to collect environmental indicators from air, water, and soil in cities B, K, and W. For air quality assessments, high-precision particulate sampling instruments, including suspended particles (PM2.5, PM10), NO_2_, and O_3_ concentration instruments, are utilized. In assessing heavy metal concentrations, dissolved oxygen content, pH levels, and urban noise levels in the water bodies of Beijing, Kunming, and Wuxi, the study first randomly selects sampling points in each city considering the differences in water body types and urban environmental characteristics using geographical information systems and environmental survey data. For sampling of water body heavy metal concentrations and dissolved oxygen content, standard water sample bottles and dissolved oxygen meters are used for collection and analysis, while parameters such as water temperature and oxidation–reduction potential are simultaneously recorded. Real-time monitoring and recording of pH levels are conducted using standard pH meters. A sampling of urban noise levels utilizes professional acoustic monitoring equipment spectrum analyzers to monitor and record noise levels in different time periods and areas. After sampling, samples are labeled, sealed, and transported for rigorous laboratory analysis and data processing. The accuracy and reliability of the analysis results are ensured through the standard curve method and monitoring of quality control samples.

### 2.3 Selection and standards of experimental data materials

Urban multi-media environmental variables encompass 10 parameters, incorporating factors such as PM2.5, NO_2_, PM10, and O_3_ concentration in the air, as well as the concentration of heavy metals in water. Elaborative information is presented in Table 2.

**Table 2:** Urban multi-media environmental factors.

Number	Variable classification	Input variables	Reason
1	Air quality	PM2.5	PM2.5 refers to fine PM in the air and is closely associated with respiratory and cardiovascular diseases. It serves as a crucial indicator for assessing urban air quality.
2		NO_2_	NO_2_, a major component of vehicle exhaust, is also linked to respiratory system diseases.
3		PM10	PM10, atmospheric PM, is similarly related to respiratory diseases and serves as a significant indicator for measuring air quality.
4		O_3_ concentration in the air	O_3_, an essential gas in the atmosphere, poses a threat to the human respiratory system at high concentrations and interacts with other pollutants. Therefore, it is an important component of air quality assessment.
5	Water quality	Heavy metal concentration in water	Heavy metals are one of the primary factors contributing to water pollution, directly impacting water quality. Prolonged exposure may pose health risks to humans.
6		Dissolved oxygen content in water	Dissolved oxygen is a critical biological indicator in water bodies, influencing the balance of aquatic ecosystems and having certain effects on both aquatic organisms and human health.
7		pH value in water	The pH value reflects the acidity or alkalinity of water and is related to the water’s acid–base balance and pollution levels. It is a crucial parameter for assessing water health.
8	Soil quality	Organic matter content in the soil	High levels of organic compounds may lead to soil pollution, directly affecting the safety of agricultural products and water sources. Therefore, it is an important indicator in environmental monitoring.
9		Nitrogen and phosphorus content in soil	Nitrogen and phosphorus in soil serve as indicators of agricultural non-point source pollution directly related to agricultural production and water quality. They are closely associated with the comprehensive assessment of urban environmental impacts.
10	Noise level	City noise level	Noise is a form of pollution in urban environments. Prolonged exposure may negatively impact hearing, sleep, and mental health. It is a crucial factor in the comprehensive assessment of urban environmental impacts.

This study refers to data indicator standards released by the World Water Quality Portal (WQP), World Air Quality Index (WQI), International Soil Reference and Information Centre (ISRIC), and OpenStreetMap (OSM). The specific details of the datasets are provided in Table 3.

**Table 3:** Dataset required for experiments.

Dataset	Dataset description	The amount of data
WQP	Water quality data set	1,582
WQI	Air quality data set	1,448
ISRIC	Soil dataset	1712
OSM	Noise level data set	1,615

### 2.4 Indicators for research and verification

The core of this study lies in accurately assessing the impact of the urban environment on residents’ health. Considering the categorical characteristics of selected variables in the study, in multimedia environmental monitoring, health issues related to residents include both environmental and sensory factors. Therefore, this study selects three categories of indicators: Indoor Air Quality Index (IAQI), Thermal Comfort Index (TCI), and Noise Pollution Index (NPI) to conduct a significant analysis of the urban health risk assessment model on major health indicators. IAQI is a comprehensive index used to evaluate air quality by quantifying the concentrations of various pollutants in the air and mapping them to graded standards within a certain range to reveal the levels of air quality and their impact on human health. IAQI assesses air quality by mapping monitored air pollutant concentrations to predefined graded intervals and combining the index ranges of the graded intervals. It uses linear interpolation to convert the monitored concentrations into corresponding indices, thereby reflecting the level of air quality. The specific calculation of IAQI is shown in Equation (1):


(1) $$\mathrm{IAQI}=\frac{\left(I_{\mathrm{high}}-I_{\mathrm{low}}\right)\times \left(C-C_{\mathrm{low}}\right)}{\left(C_{\mathrm{high}}-C_{\mathrm{low}}\right)}+I_{\mathrm{low}}$$

In Equation (1), C represents the monitored air pollutant concentration; $C_{\mathrm{low}}$ and $C_{\mathrm{high}}$ represent the lower and upper limits of pollutant concentration graded intervals, respectively; $I_{\mathrm{low}}$ and $I_{\mathrm{high}}$ represent the corresponding lower and upper limits of the index ranges of the graded intervals.

TCI serves as a comprehensive indicator for assessing human comfort, mainly describing the impact of environmental temperature and humidity on the human body. TCI considers factors such as environmental temperature, humidity, and wind speed. This index can reflect the degree of thermal comfort perceived by the human body, as well as the physiological and psychological effects of environmental conditions on the human body. In the calculation process of this study, the effect of temperature on thermal comfort is represented by T−14.3 , and wind speed is corrected by $0.0216\cdot V\cdot \left(T-14.3\right)$ . The effect of relative humidity is presented in the form of $\left(1-0.01\;\cdot \;|70.5-RH|\right)$ . Considering these factors, TCI reflects the impact of environmental conditions on human thermal comfort, with higher values indicating greater comfort. The calculation of TCI is shown in Equation (2):


(2) $$TCI=\left(\frac{T-14.3}{1+0.0216\cdot V\cdot \left(T-14.3\right)}\right)+\left(1-0.01\;\cdot \;|70.5-RH|\right)$$

In Equation (2), T represents environmental temperature; V represents wind speed; RH represents relative humidity.

NPI is a comprehensive index used to measure the level of noise pollution in the environment, mainly based on acoustic principles and environmental noise monitoring data. NPI considers factors such as the frequency, intensity, duration, and spatial–temporal distribution of noise, calculated through mathematical models. This index can reflect the degree of impact of environmental noise on human health and quality of life, as well as its impact on the ecological systems of animals and plants.


(3) $$NPI=\frac{\sum_{i=1}^{n}\left(p_{i}\times d_{i}\right)}{\sum_{i=1}^{n}p_{i}}$$

In the calculation process of NPI, it is based on the weighted average of the contribution rates of each noise source and the decibel level. Each term in the formula represents the contribution of a noise source, and the comprehensive NPI is obtained by summing the weighted contributions of all noise sources. $p_{i}$ represents the contribution rate of the noise source i ; $d_{i}$ represents the decibel level of noise source i .

In order to ascertain the model’s applicability across diverse urban settings, this study conducts a comparative analysis of assessment outcomes across multiple cities. Specific applicability indicators are employed to assess the model’s robustness. The delineated applicability indicators are detailed in Table 4.

**Table 4:** Indicators for suitability analysis.

Index	Specific Discussion	Significance
Root Mean Square Error (RMSE)	It is a measure of the average deviation between the model’s predictions and the actual observations.	The smaller the RMSE value, the higher the prediction accuracy of the model and the better the fit to the actual observed values.
Coefficient of determination (*R*^2^)	This coefficient reflects the degree to which the model explains the variability of the dependent variable.	*R*^2^ close to 1 indicates that the model better explains the variation of the observed data and the closer it is to a perfect fit.
Residual term	It refers to the difference between model predictions and actual observed values.	The size and distribution of the residual terms reflect the model’s fit to different observations. Small residuals indicate that the model fits the data well.

## 3 Fuzzy intelligent computing model based on FIS

### 3.1 Basic principles of FIS

The FIS constitutes a computational model grounded in fuzzy logic theory, specifically devised to adeptly handle information characterized by uncertainty and vagueness (21, 22). The implementation of FIS involves integrating fuzzy rule bases, fuzzy sets and membership functions, and fuzzy inference engines to handle uncertainty and fuzziness in events. The fuzzy rule base is constructed by expert knowledge, specifying the fuzzy relationships between inputs and outputs. Fuzzy sets and membership functions define the fuzzy characteristics of input and output variables, fuzzifying them to adapt to real-world situations. Finally, through the fuzzy inference engine, FIS performs fuzzy reasoning based on the fuzzy rule base and fuzzy inputs, mapping fuzzy inputs to fuzzy outputs according to the logical relationships between fuzzy rules and input values (23–25). FIS combines fuzzy logic and inference to handle various complex nonlinear systems and exhibits good performance and interpretability in uncertain environments (26).

**Figure 1:**
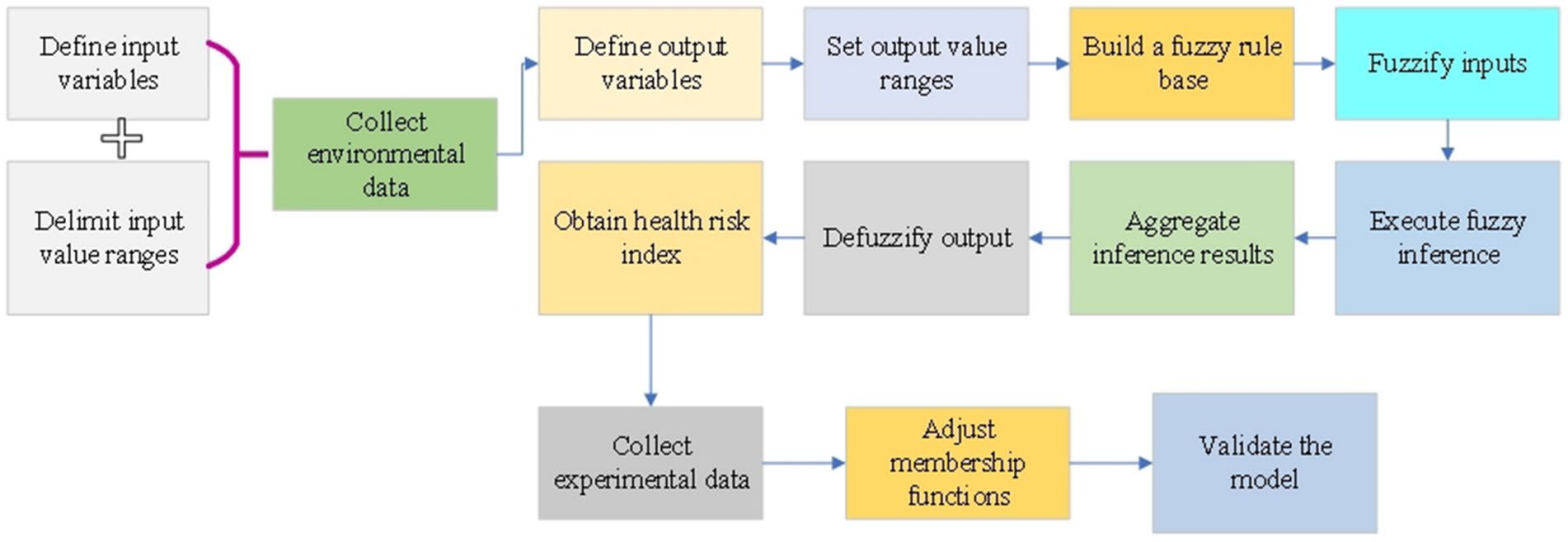
FIS implementation process.

In the context of this study, the FIS serves the purpose of amalgamating multi-media environmental monitoring data for the assessment of urban health risks. By emulating the fuzzy reasoning process inherent in human cognition, the system adeptly manages the fuzzy relationships inherent in diverse environmental factors, thereby furnishing a comprehensive evaluation of health risks (27, 28). This approach facilitates a nuanced understanding of the precise impact of urban environments on resident health, culminating in comprehensive evaluation results (29, 30). The specific procedural intricacies are delineated in Figure 1.

### 3.2 Computational process of the FIS

In order to articulate the membership of input variables, the FIS employs membership functions (31, 32). This function accepts the variable value as input and employs a designated curve to depict the extent of membership of the variable to a specific fuzzy set, as exemplified in Equation (4):


(4) $$\mathrm{\mu High}:A\left(x\right)=\frac{1}{1+\exp\left(-\frac{c_{\mathrm{High}:A}*\left(x-a_{\mathrm{High}:A}\right)}{b_{\mathrm{High}:A}}\right)}$$

In Equation (4), *A* represents the input variable; *x* denotes the variable value; $a_{\mathrm{High}:A}$ signifies the center of the input variable’s membership function; $c_{\mathrm{High}:A}$ is the parameter governing the steepness of the control curve; $b_{\mathrm{High}:A}$ corresponds to the base of the changing curve.

Within the FIS, the membership functions associated with fuzzy output variables assume a pivotal role. These membership functions portray the membership of the output variable across various risk levels through triangular-shaped curves (33, 34). This functional form facilitates the establishment of transparent associations within the fuzzy set of output values, effectively translating the fuzzy information of the input into fuzzy results for the output, as exemplified in Equation (5):


(5) $$\mathrm{\mu Medium}:C\left(y\right)=\exp\left(-\frac{{\left(y-b_{\mathrm{Medium}:C}\right)}^{2}}{2{\sigma^{2}}_{\mathrm{Medium}:C}}\right)$$

In Equation (5), $\sigma_{\mathrm{Medium}:C}$ denotes the standard deviation, and $b_{\mathrm{Medium}:C}$ signifies the center of the membership function associated with the output variable (35).

Within the FIS, the ultimate output undergoes a defuzzification process utilizing designated methods, transforming the membership function of the fuzzy output into a distinct, singular value (36), as exemplified in Equation (6):


(6) $$\mathrm{Output}=\frac{\sum_{i=1}^{n}\left(w_{i}*\mathrm{Outpu}t_{i}\right)}{\sum_{i=1}^{n}w_{i}}$$

In Equation (6), $\mathrm{Outpu}t_{i}$ represents the output of the *i*-th rule, and $w_{i}$ represents the weight of the corresponding rule.

Furthermore, considering that the comprehensive index used in the referenced city data adopts unified values published by the Chinese Ministry of Ecology and Environment, the calculation process for the comprehensive index in this study also follows the corresponding calculation method. The calculation of the comprehensive index is shown in Equation (7):


(7) $$I_{\mathrm{sum}}=\underset{p}{\sum }\frac{C_{p}}{S_{p}}$$

In Equation (7), $I_{\mathrm{sum}}$ represents the comprehensive index of environmental air quality. $C_{p}$ represents the concentration value of pollutant *p*. When p is SO_2_, NO_2_, PM10, and PM2.5, $C_{p}$ is the monthly average. When p is CO and O_3_, $C_{p}$ is the concentration value at a specific percentile. $S_{p}$ represents the annual average secondary standard for pollutant p . When p is CO, it represents the daily average secondary standard. When p is O_3_, it represents the 8-h average secondary standard.

## 4 Analysis of results on urban multi-media environmental monitoring and health risk assessment with the introduction of fuzzy intelligent computing model

### 4.1 Credibility assessment analysis

**Figure 2:**
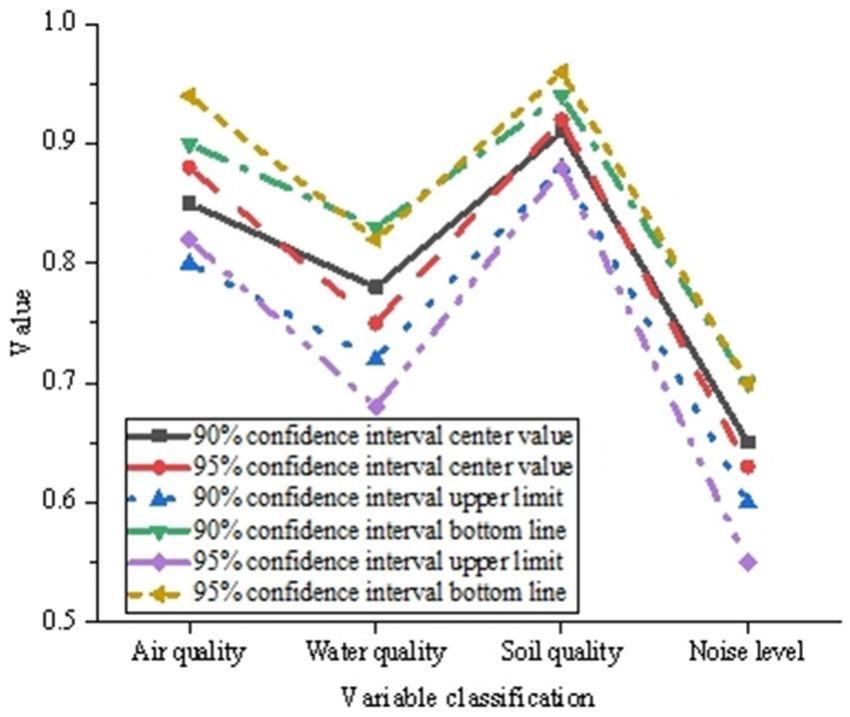
Credibility evaluation results of the constructed urban health risk assessment model.

Establishing confidence intervals at varying confidence levels facilitates a more exhaustive comprehension of the stability and credibility associated with model outputs. The analysis of credibility assessment contributes to a more thorough and precise interpretation of the model’s applicability and reliability in health risk assessment. The results of the credibility assessment are depicted in Figure 2.

In Figure 2, under diverse urban and environmental conditions, the model’s estimation of the comprehensive health risk index exhibits a discernible credibility range. Within the 90% confidence interval, the model’s health risk estimation demonstrates relative stability, characterized by a narrow confidence interval. Conversely, within the 95% confidence interval, the interval widens, signaling a more exhaustive consideration of uncertainty in the model output. This variation suggests that at higher confidence levels, the confidence interval broadens, enhancing confidence in the model output, albeit with a proportional increase in the uncertainty of the estimation. In essence, the model is adept at furnishing credibility ranges at distinct confidence levels during health risk assessments, thereby contributing to a more comprehensive grasp of the reliability inherent in the model output.

### 4.2 Significance analysis results

**Figure 3:**
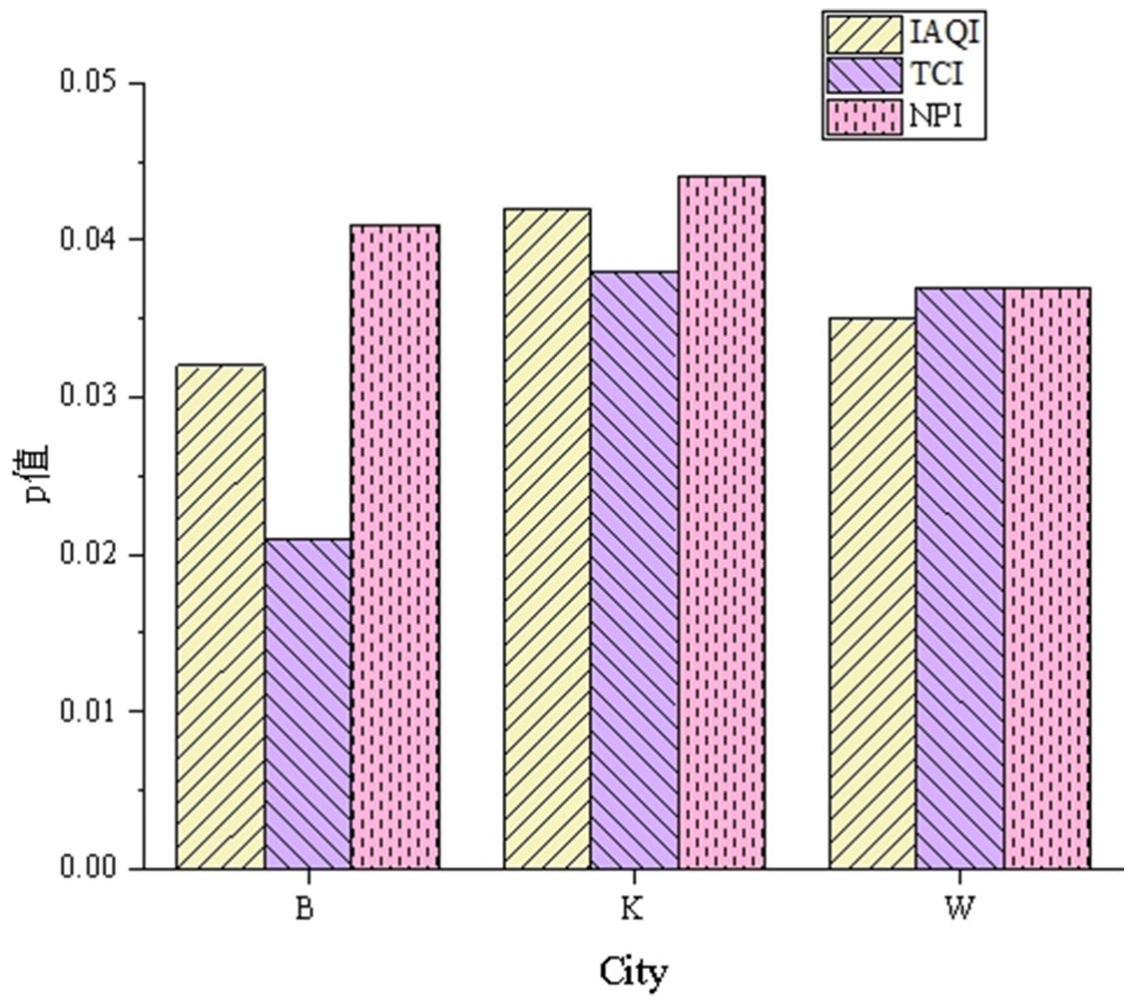
Significance analysis results of the urban health risk assessment model for major urban health indicators.

The IAQI can directly reflect the potential impact of pollutants in the air on human health, and the TCI can evaluate the impact of the thermal environment on residents’ comfort. The NPI focuses on the degree of interference from noise pollution in urban areas. The significance analysis results are shown in Figure 3.

In Figure 3, under different urban and environmental conditions, the model’s *p*-values for the three core indicators IAQI, TCI, and NPI all satisfy *p* < 0.05. For City B, the p-values for IAQI, TCI, and NPI are 0.032, 0.021, and 0.041, respectively. In City K, the *p*-values for all three indices are around 0.041, while in City W, the p-values for all three indices range from 0.035 to 0.037. The data indicate that the model’s evaluation results for the three representative urban health indicators are statistically significant. This implies that the model has strong predictive capabilities under different urban environments and possesses a certain degree of accuracy in assessing the health risks of urban residents.

### 4.3 Correlation results analysis

**Figure 4:**
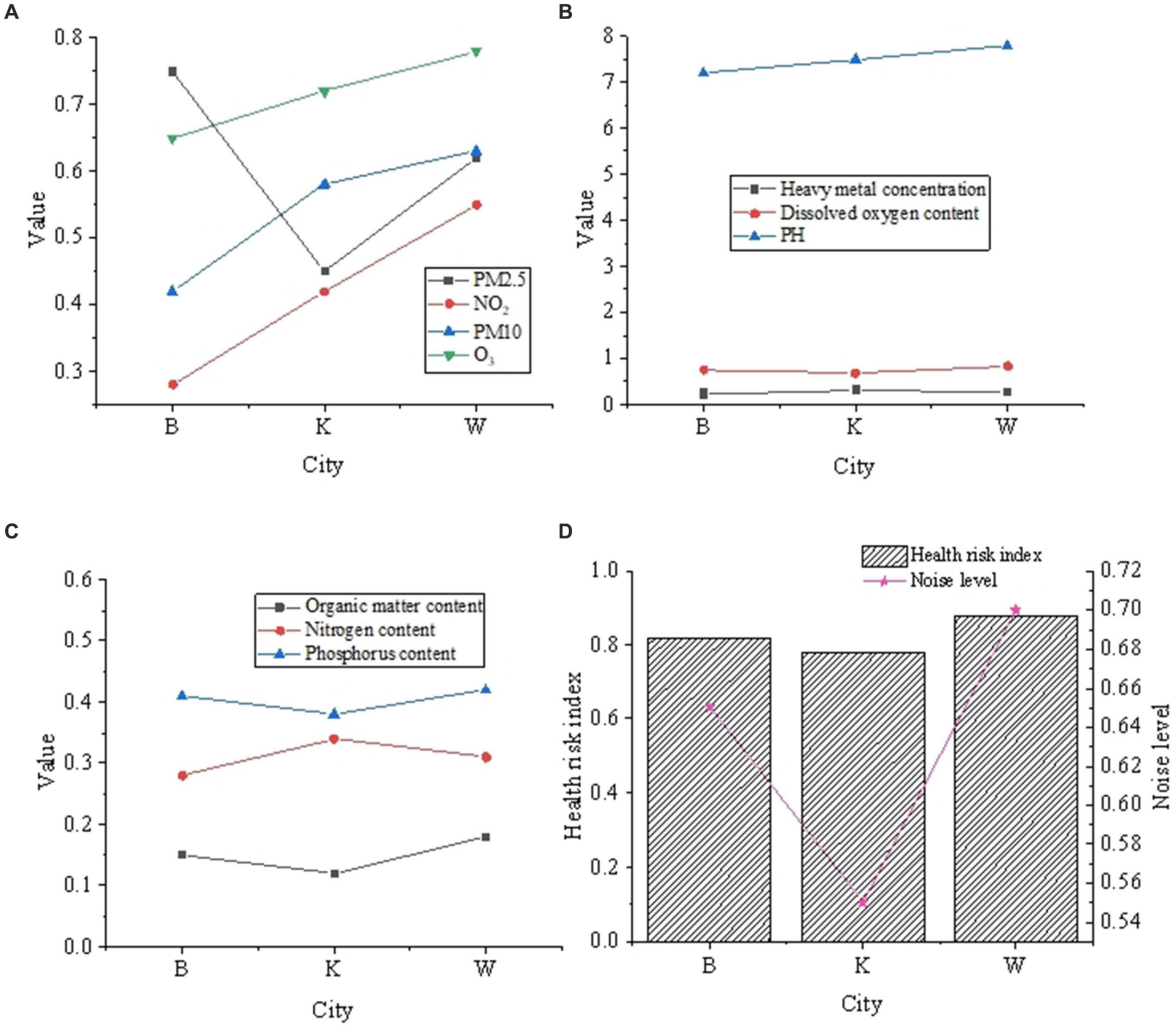
Correlation analysis results between different urban environmental factors and health risk indexes. **(A)** Air quality factors. **(B)** Water quality related factors. **(C)** Soil quality factors. **(D)** Noise factors and risk assessment calculation.

Correlation analysis delves into the intricate relationship between diverse urban environmental factors and the health risk index. Through specific correlation coefficients, it becomes feasible to preliminarily discern the weights of various factors in different cities. The outcomes of the correlation analysis are delineated in Figure 4.

In Figure 4, within City *B*, a robust positive correlation is evident between PM2.5 concentration and the health risk index, represented by a correlation coefficient of 0.75. This signifies that an escalation in PM2.5 concentration corresponds to an increased health risk for residents in City *B*. Concurrently, noise levels also exhibit a positive correlation with the health risk index (correlation coefficient of 0.65), suggesting that noise similarly exerts a discernible impact on the health of City *B*’s residents. In City *K*, the correlation between PM2.5 concentration and the health risk index is relatively modest (correlation coefficient of 0.45), whereas the correlation between noise levels and the health risk index is more pronounced (correlation coefficient of 0.55). This data implies that in an environment characterized by relatively clean air, noise may exert a more substantial influence on the health of residents. Within City *W*, the positive correlation between PM2.5 concentration and the health risk index is moderate (correlation coefficient of 0.62). In comparison to City *B* and City *K*, City *W* exhibits the highest positive correlation between noise levels and the health risk index (correlation coefficient of 0.7), indicating that noise plays a pivotal role in health-related concerns for residents in City *W*. Generally, the impact of PM2.5 concentration on health is discernible across all three cities, while noise levels exert a more pronounced effect in Cities *K* and *W*.

Specifically, the impact of PM2.5 concentration on urban environmental monitoring varies significantly across different cities. In City *B*, the strong positive correlation with high PM2.5 concentration indicates poor air quality, elevating the risk of residents developing respiratory and cardiovascular diseases. The positive correlation with noise levels further highlights the potential threat of urban noise to health. In City *K*, within a relatively clean air environment, the heightened correlation between noise levels and health risks underscores the independent impact of noise on health, potentially leading to issues like sleep disorders. Meanwhile, in City *W*, despite moderate PM2.5 concentration, noise levels emerge as a primary factor, reflecting the intricate relationships within the urban environment where diverse factors interweave and influence human life and health. The environmental factors contributing to health risks vary across different cities, necessitating tailored environmental protection and health intervention measures based on specific circumstances.

### 4.4 Applicability results analysis

**Figure 5:**
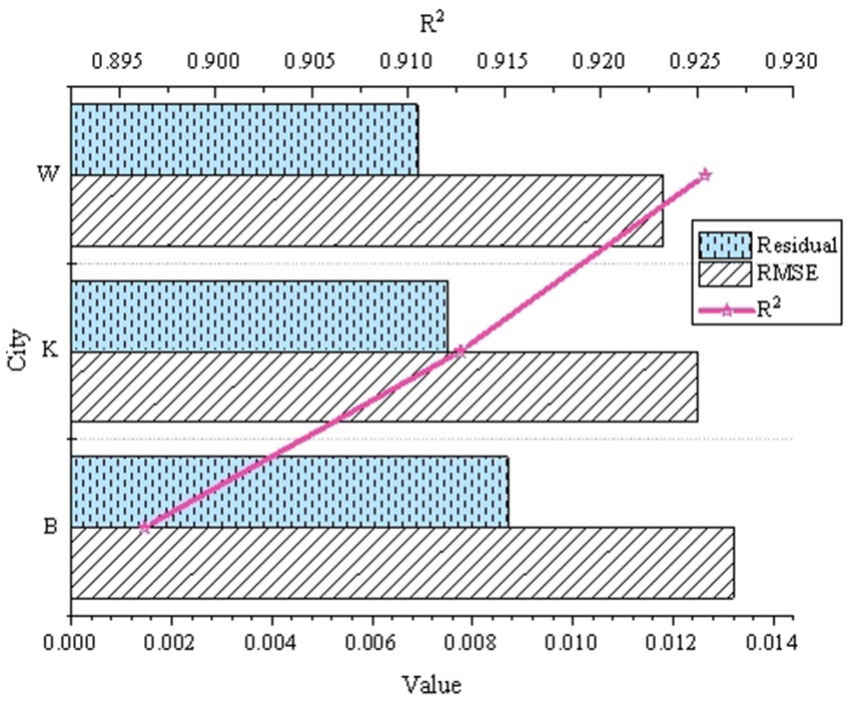
Analysis results of the applicability of the urban health risk assessment model under different urban and environmental conditions.

Applicability analysis endeavors to affirm the viability of the model across diverse urban and environmental conditions. Through a comparative examination of data indicators across three cities, a holistic comprehension of the model’s efficacy in various environmental contexts is attained, thereby assessing its generalization capability and robustness. The outcomes are elucidated in Figure 5.

In Figure 5, the RMSE values are diminutive, registering at 0.0132, 0.0125, and 0.0118, respectively. This data signifies that the model displays a high degree of accuracy in prediction across the three cities, aligning closely with the observed values. The *R*^2^ values stand at 0.8963, 0.9127, and 0.9254, nearing 1, indicating that the model effectively elucidates variations in the data and possesses robust explanatory capability. The relatively modest magnitudes of the residual terms, measuring 0.0087, 0.0075, and 0.0069, imply minimal disparities between the model’s predicted outcomes and the observed values, showcasing a relatively even distribution of residuals. Consequently, this model manifests notable robustness and adaptability across diverse environmental conditions, facilitating the assessment of health risks in assorted urban settings.

## 5 Discussion

This study presents two key innovations in the field of environmental health risk assessment. Firstly, the study employs multi-media environmental monitoring data for urban health risk assessment. By utilizing key indicators such as PM2.5, NO_2_, PM10, O_3_, and others as inputs, coupled with a comprehensive health risk index as the output, it achieves a holistic evaluation of urban environments. Compared to single-media assessment methods, this approach offers higher scientific rigor and practical utility. This integrated assessment across various media provides a more comprehensive and authentic reflection of the diverse health threats residents face. Secondly, the study analyzes regional characteristics by selecting cities with distinct air quality features—Beijing (high pollution), Kunming (low pollution), and Wuxi (moderate pollution)—to evaluate model performance. This regional analysis highlights the impact of geographical variations on health risks, which is particularly relevant to China’s diverse environmental conditions. The *R*^2^ values are 0.8963, 0.9127, and 0.9254, respectively, approaching 1. Additionally, the model’s *p*-values for the three core indicators IAQI, TCI, and NPI satisfy *p* < 0.05 under different urban environments. The data indicate that the model provides a comprehensive explanation for the variability in the data and possesses strong explanatory power.

However, the study acknowledges limitations, including the lack of consideration for individual differences and the long-term health impacts of environmental exposure. Future research will address these gaps by incorporating more precise environmental monitoring data and exploring individual variability factors. The significance of this study lies in its refinement of the relationship between urban environments and health, providing innovative approaches for urban environmental health assessment and enhancing assessment accuracy. This research contributes to the development of more effective strategies for environmental protection, cleaner production, and sustainable urban planning.

## 6 Conclusion

This study employs a Fuzzy Inference System (FIS) to assess urban health risks through the integration of multi-media environmental monitoring data, including PM2.5, NO_2_, PM10, and O_3_. The model’s effectiveness is demonstrated through credibility, correlation, and applicability analyses. City-specific analyses unveil notable findings. In Beijing (*B* City), a strong positive correlation is observed between PM2.5 concentration and the health risk index (correlation coefficient 0.75), with noise levels also showing a positive correlation (correlation coefficient 0.65). Kunming (*K* City), with cleaner air, exhibits a greater impact of noise on health, while Wuxi (*W* City), with moderate pollution, shows positive correlations for both PM2.5 and noise levels (correlation coefficients 0.62 and 0.7, respectively). The model’s minimal residual values and uniform distribution indicate exceptional robustness and adaptability, providing reliable support for urban health risk assessment. This research underscores the importance of multidimensional environmental data in developing comprehensive and sustainable urban health management strategies.

## Data availability statement

The original contributions presented in the study are included in the article/supplementary material, further inquiries can be directed to the corresponding author.

## Author contributions

WW: Conceptualization, Data curation, Resources, Writing – review & editing. XG: Investigation, Methodology, Writing – original draft. XP: Data curation, Formal analysis, Methodology, Project administration, Writing – review & editing. ZW: Conceptualization, Formal analysis, Supervision, Writing – review & editing. XL: Validation, Visualization, Writing – original draft. JZ: Investigation, Software, Writing – original draft.
